# Renoprotective effect of *Limonium duriusculum* (de Girard) Kuntze via modulation of oxidative stress/ UPR markers and inflammation during cyclosporine-induced nephrotoxicity in rats

**DOI:** 10.22038/IJBMS.2024.77052.16661

**Published:** 2024

**Authors:** Azzedine Redouane-Salah, Ameddah Souad, Wafa Kerkatou, Kamil Wojnicki, Adrian M. Ramos, Alberto Ortiz, Bozena Kaminska, Ahmed Menad

**Affiliations:** 1 Laboratoire de Biologie et Environnement, Faculté des Sciences de la Nature et de la Vie, Université Frères Mentouri Constantine 1, Route Aïn El Bey, 25000, Constantine, Algérie; 2 Unité de Recherche, Valorisation des Ressources Naturelles, Molécules Bioactives et Analyses Physicochimiques et Biologiques (VARENBIOMOL), Université Frères Mentouri, Constantine 1, Route Aïn El Bey, 25 000 Constantine, Algérie; 3 Laboratory of Molecular Neurobiology, Nencki Institute of Experimental Biology, Warsaw, Poland; 4 Department of Nephrology and Hypertension, IIS-Fundacion Jimenez Diaz UAM, Madrid, Spain

**Keywords:** Cyclosporine, ER stress, Inflammatory markers, Limonium duriusculum, Nephrotoxicity, Oxidative stress

## Abstract

**Objective(s):**

The present study aimed to explore the mechanisms underlying the potency of the renoprotective effect of the EtOAc fraction of *Limonium duriusculum* (EALD) (Plumbaginaceae) against cyclosporine A (CsA), in comparison to vitamin E (Vit. E).

**Materials and Methods:**

In the *in-vivo* experiment, a model of CsA-induced nephrotoxicity was established by dosing male Wistar rats with 25 mg/kg, for 14 days. The protective effect of EALD was investigated through pretreatment of rats with a dose of 200 mg/kg for 14 days, compared to the oral administration of Vit. E at 100 mg/kg. Renal function and markers of oxidative stress were then assessed. Furthermore, a complementary *in-vitro* study was carried out to evaluate CsA-induced endoplasmic reticulum stress (ERS) and inflammation on cell culture (3T3 cells and MCT cells) using western blot and quantitative RT-PCR..

**Results:**

Pretreatment of rats with EALD significantly attenuated the elevated levels of renal dysfunction markers (BUN, creatinine) and suppressed malondialdehyde (MDA) levels; It also significantly regulated the changes in superoxide dismutase (SOD), reduced glutathione (GSH), glutathione peroxydase (GPx), and glutathione S-transferase (GST) levels as compared to Vit. E, demonstrating a more effective recovery in renal tissues. Treatment of cells with CsA was linked to the expression of ERS and inflammatory markers activating transcription factor (ATF4), inositol-requiring enzyme 1α (IRE1α), binding immunoglobulin protein (BiP), and monocyte chemoattractant protein-1 (MCP1). In contrast, pretreatment of cells with EALD resulted in a significant decrease in both ERS and inflammatory markers.

**Conclusion:**

These findings indicate the renoprotective potential of *L. duriusculum*, as it demonstrated the ability to ameliorate CsA-induced renal dysfunction through its distinctive antioxidant properties.

## Introduction

Cyclosporine A (CsA) is an effective immunosuppressive drug commonly used to prevent allograft rejection in solid organ transplants as well as to treat various inflammatory and autoimmune diseases. However, several side effects from long-term administration of CsA have been reported, with nephrotoxicity being the most significant (1). Two forms of nephrotoxicity have been identified: an acute reversible form characterized by renal vasoconstriction and an irreversible chronic form characterized by glomerular vasoconstriction and structural changes, including arteriopathy, and tubulointerstitial fibrosis (2). At present, the cellular and molecular mechanisms of CsA nephrotoxicity are not entirely understood, although the involvement of reactive oxygen species (ROS) has received significant attention (3) which subsequently induces various degrees of cell damage including inflammation, apoptosis, endoplasmic reticulum stress (ERS) and autophagy (4, 5). ERS and oxidative stress are interconnected in their responses to physiological and pathological stressors. With the persistence of both ERS and oxidative stress, activation of the unfolded protein response (UPR) becomes inevitable to maintain cell function and survival, otherwise, apoptotic pathway processes are initiated (6). Furthermore, the UPR is linked to inflammation on several levels leading to cytokine production and the modulation of inflammatory signaling pathways (7). Notably, both past and recent research have shown that ER stress is implicated in the progression of glomerular and tubular dysfunction in various renal diseases, including CsA nephrotoxicity (8). However, further clarification is needed regarding the involvement of ERS in kidney damage transition. Typically, inhibiting oxidative stress results in a reduction in ERS, decreasing UPR activation and yielding improved outcomes (9, 10). In this regard, the use of different antioxidants or free radical scavengers, such as vitamin E, vitamin C, melatonin, and plant polyphenols like quercitin and catechin as well as the minerals selenium and zinc as a therapeutic strategy against CsA-induced nephrotoxicity has been largely demonstrated (11-13). Despite the few positive results of pharmacological inhibition of ERS during nephrotoxicity, it potentially causes various side effects, whereas the use of natural products as a pretreatment or co-treatment remedy for various diseases, especially those related to ER disorders, has proven to be safer and more effective (14).

*Limonium* is a halophyte plant that often grows in arid or semi-arid plains and coasts, where it resists drought and salinity. This genus belongs to the *Plumbaginaceae* family, represented by about 150 wild species worldwide (15) and 23 species in Algeria (16). Many species of *Limonium *were commonly used in traditional medicine as a cure to treat various organic diseases and disorders, such as allergies, fever, and arthritis (17, 18). Several published studies showed that bioactive compounds of *Limonium* can protect the liver and heart (19, 20) and possess anti-inflammatory, antiviral, and anti-cancer actions (21-23). *Limonium duriusculum* (de Girard) Kuntze is utilized by the local population for the treatment of allergies, as indicated in a previous study that highlighted its noteworthy bioactive properties, especially in terms of antioxidant activity (17). Our previous studies clearly reported the antioxidant activity of EALD, such as DPPH° scavenging activity, iron chelating capacity, and lipid peroxide inhibition (17).

Given that the EtOAc fraction from *L. duriusculum *(EALD)(the East of Algeria MILA region; Algeria) has not previously undergone screening for its protective effect against renal injury. The present study investigates the underlying mechanisms of EALD, particularly the potential involvement of antioxidants, anti-inflammatory properties, and the effectiveness of ERS modulation in preserving renal structure and function during CsA-induced nephrotoxicity in a rat model through a combination of *in vitro* and *in vivo* experiments.

## Materials and Methods

***Chemicals and reagents***

Cyclosporine, Neoral^®^ (SANDIMMUN *NEORAL* caps 100 mg) was obtained from Novartis Pharma  (Basel, Switzerland)*,* cyclosporine used for cell culture experiments was obtained from Calbiochem (La Jolla, CA, USA), 5,5′-dithio-bis-2-nitrobenzoic acid (DTNB),1-chloro-2,4-dinitrobenzene (CDNB), reduced glutathione (GSH), glutathione-S-transferase (GST), glutathione peroxidase (GPx), superoxide dismutase (SOD), trichloroacetic acid (TCA), thiobarbituric acid (TBA), malondialdehyde (MDA), Carazzi’s hematoxylin solution, 3,3’-diaminobenzidine (DAB), protease inhibitor, and all other buffers and chemicals used in experiments were purchased from Sigma-Aldrich Co. (St. Louis, MO, USA). RPMI 1640 (GIBCO, Grand Island, NY.USA), glutamine (2 mM), penicillin, streptomycin, fetal bovine serum (FBS), decomplemented fetal bovine serum (DFBS), Dulbecco’s Modified Eagle’s Medium (DMEM)(Sigma Aldrich, Germany), sodium citrate buffer; primary antibody: polyclonal rabbit anti-rat IRE1α (1/350, ab48187Abcam Inc. UK), goat anti-rabbit IgG H&L (HRP)(1/250, ab205718Abcam Inc. UK) was used as a secondary antibody, Plasma Membrane Extraction Kit (MBL International Corporation. USA); primary antibodies anti-inositol-requiring enzyme 1α (IRE1α), Anti-Binding immunoglobulin protein (BiP/GRP78), secondary antibodies (Molecular Probes, Invitrogen, Carlsbad, CA, USA), High Capacity cDNA Archive Kit, (Applied Biosystems, Foster City, CA), Pre-developed primer and probe assays were all from Applied Biosystems, All other chemicals and reagents used were of analytical grade.

***Plant material and extract preparation***

According to Quezel and Santa (16), aerial parts of *L. duriusculum* (de Girard) were collected (May 2018) in the region of Mila in North-East Algeria and confirmed by Professor HocineLaouer (Ferhat Abbas University, Setif, Algeria). A voucher specimen (LDP0510-MIL-ALG-66) was placed at the Herbarium of the VARENBIOMOL research unit, Frères Mentouri University, Constantine 1.

The dried leaves and flowers of *L. duriusculum* (2000g) were macerated in a hydro-alcoholic solution MeOH/H_2_O (70:30, v/v, 24 hours) three times at ambient temperature. The filtrates were mixed, concentrated under decreased pressure (up to 35 ^°^C), dissolved in H_2_O with magnetic stirring, and kept at 4 ^°^C overnight to precipitate as much chlorophyll as possible. Following filtering, the resultant solution was extracted with chloroform, ethyl acetate, and n-butanol. The organic phases were dried with Na_2_SO_4_, filtered with standard filter paper, and concentrated in a vacuum at room temperature to provide the following extracts: CHCl_3_ (2.1 g), EtOAc (22.02 g), and *n*-BuOH (29.8 g). A part of EtOAc extracts (EALD) was selected for the current nephroprotective study.

***In vivo experimental***

Male Wistar rats weighing 200-250 g were used in the experiment. Animals were randomized into four groups of six animals, each housed in cages and kept at a controlled room temperature (23±2 ^°^C) under a 12 hr light/dark cycle and fed standard feed and water The *in vivo* experimental protocol was authorized by the Research Program Committee (PRFU, D01N01UN250120190002). which were in strict compliance with the United States National Institutes of Health Guidelines for care and use of laboratory animals in biomedical research.

***Animals and experimental design***

• Group I: (Control): Rats served as the control group, treated orally with olive oil as a vehicle for 14 days.

• Group II: (EALD): Rats treated orally with EALD dissolved in distilled water (200 mg/ kg/day) for 14 days.

• Group III: (CsA): Rats received daily oral CsA (25 mg/kg/day)(24) dissolved in olive oil for 14 days.

• Group IV: (EALD+CsA): Rats treated orally with EALD dissolved in distilled water (200 mg/ kg/day) and co-treated with CsA (25 mg/kg/day) dissolved in olive oil for 14 days. 

• GroupV: (Vit.E+CsA) as positive control: Rats treated orally with Vitamin E (100 mg/kg/day)(24) and co-treated with CsA (25 mg/kg/day) dissolved in olive oil for 14 days.

***Biochemical estimation***

At the end of the *in vivo *experimental phase (day 15), blood samples were collected in heparinized tubes through the retro-orbital venous plexus and centrifuged at 3000 rpm for 15 min at 4 ^°^C; the plasma was stored at 4 ^°^C to estimate renal function markers: blood urea nitrogen (BUN) and plasma creatinine. These estimates were carried out according to the usual protocols provided with the analysis kits.

Under ether anesthesia, the animals were sacrificed by cervical dislocation. The kidneys were promptly removed, weighed, and rinsed with ice-cold saline (0.9%) before being cut into pieces for examination and analysis.

***Preparation of kidney homogenate***

Renal cortex tissue was rapidly homogenized in ice-cold phosphate-buffered saline (PBS) (0.1 M pH=7.4). The homogenate (10%, w/v) was centrifuged at 9600×g for 45 min at 4 ^°^C to separate cytosolic fraction. The obtained supernatant would then be used for the assessment of MDA and oxidative stress markers (SOD, GSH, GPx, and GST). The total protein concentration was evaluated by the Lowry method (25).

***Assessment of renal oxidative stress Markers***

*Determination of malondialdehyde (MDA)*

The level of MDA was evaluated based on the generation of thiobarbituric acid reactive substances (TBARS), which can be measured by exploiting the optical density value of the supernatant at 532 nm according to the method of Ohkawa *et al. *(26). The results were expressed in nmol MDA g^-1 ^of tissue.

***Measurement of superoxide dismutase activity (SOD)***

SOD activity in kidney tissue was determined by measuring the inhibition of pyrogallol auto-oxidation, following the technique described by Marklund and Marklund (27).

One unit of enzyme activity was defined as the amount of enzyme causing 50% inhibition of pyrogallol autoxidation per minute under the assay conditions.

***Determination of reduced glutathione level (GSH)***

The level of GSH in kidney tissue was determined colorimetrically by reacting GSH with Ellman’s reagent according to the method described by Sedlák and L’ Hanus (28). Absorbance was measured at 412 nm, and GSH values ​​were expressed as nmol GSH/mg protein.

***Determination of glutathione S-transferase Activity (GST)***

GST activity was quantified based on its ability to conjugate the GSH with 1 CDNB, following the method described by Habig* et al*. (29). The GST activity was determined by monitoring changes in absorbance at 340 nm for 4 min, results were expressed as μmol/mg protein.

***Determination of glutathione peroxidase Activity (GPx)***

GPx activity was determined according to the method described by Rotruck *et al*. (30) based on the potential of the enzyme to catalyze the decomposition of hydrogen peroxide (H_2_O_2_). GPx activity was expressed in nmol/min/mg protein in the presence of GSH.

***Histopathological study***

For histological examinations, the remaining part of the kidney tissue was fixed in 10% phosphate-buffered formalin (pH 7). The samples were embedded in paraffin, then cut into 5 μm sections and stained with hematoxylin and eosin by routine techniques; possibly stained specimens were carefully examined.

***Cell culture and Treatment***

Murine fibroblast 3T3 cells (American Type Culture Collection (ATCC)) were incubated in DMEM supplemented with 10% FBS. Murine proximal tubular epithelial (MCT) cells (provided by Dr. Eric Neilson, Vanderbilt University, Nashville, TN, USA) were incubated in DMEM supplemented with 10% De-complemented FBS. Media were supplemented with 2 mML-glutamine, 100 U/ml penicillin and 10 mg/ml streptomycin; all cells were grown in 6-well culture plates at 37 ^°^C and 5% CO_2_. After 24 hr, cells were pre-treated at 70-80% confluence with different concentrations of EALD extract (5 and 25 μg/ml for 3T3 cells and 5, 25, and 50 μg/ml respectively for MCT cells) for 30 min. and then stimulated with 10 μg/ml CsA (Calbiochem, La Jolla, CA, USA) for 6 hr, two plates served as negative control (without any treatment) and positive control (treated with CsA only).

***Assessment of endoplasmic reticulum stress (ERS) and inflammation markers***

**Immunohistochemistry analysis**

*IRE1* *α* *(inositol-requiring kinase 1) marker in kidney tissue*

Immunostaining was performed on 3 μm thick renal tissue sections that were deparaffinized, and antigen was retrieved using the PT Link system (Dako Diagnostics, Barcelona, Spain) with sodium citrate buffer (10 mM) adjusted to pH 6. The endogenous peroxidase was blocked, and the sections were incubated overnight at 4 ^°^C with the primary antibody: rabbit polyclonal anti-ratIRE1α (1/350, Abcam ab48187). A goat anti-rabbit IgG H&L (HRP)(1/250, Abcamab205718) was used as the secondary antibody. Staining was performed using the Dako Autostainer (Dako Diagnostics) using 3,3′-diaminobenzidine (DAB) as a chromogen. Sections were counterstained with Carazzi’s hematoxylin.

***Western blot analysis***

*IRE1* *α* *and Binding immunoglobulin protein (BiP) expression in Murine fibroblast (3T3) cells*

Murine fibroblasts 3T3 cells were lysed in salt extraction buffer containing 0.5 M Tris, 1% NP40, 1% Triton X-100, 1 g/l SDS, 1.5 M NaCl, 0.2 M EDTA, 0.01 M EGTA, and 0.2 mmol/l protease inhibitor. The extraction of the total membrane protein fraction was obtained with the Plasma Membrane Extraction Kit, according to the manufacturer’s instructions. Membranes were stripped for 30 min at room temperature at 100 mM glycine and 20% SDS buffer, pH 3.0, washed, blocked, then probed with primary antibodies anti-inositol-requiring enzyme 1α (IRE1α) and Anti-BiP/GRP78 (78-kDa glucose-regulated protein). After incubation with the secondary antibodies (Molecular Probes, Invitrogen), immuno-complexes were detected by SuperSignal West Pico PLUS Chemiluminescent Substrate (Thermofisher Scientific, Rockford, IL, USA) and recorded on X-Ray film. Densitometric analysis was performed using Image J 1.46 software (Wayne Rasband, National Institutes of Health, USA).

***Quantitative real-time PCR analysis***

*Activating transcription factor 4 (ATF4)and Monocyte chemoattractant protein-1 (MCP-1) in MCT proximal tubule cells*

For chain reaction (qRT-PCR) analysis of the expression of pro-inﬂammatory cytokine; Monocyte chemoattractant protein-1(MCP-1), also called C-C chemokine ligand 2 (CCL2), and Activating transcription factor 4 (ATF4), 1 μg total RNA isolated from MCT cell culture using Tripure reagent (Roche, Spain) was reverse transcribed with High Capacity cDNA Archive Kit, Real-time PCR was carried out with ABI Prism 7500 Fast Real-Time PCR system (Applied Biosystems, Thermo Fisher Scientific) using the DeltaDelta Comparative CT method. RT-PCR was performed using the following pre-designed primers (Applied Biosystems, Thermo Fisher Scientific): Ccl2(Mm00441242_m1) and ATF4(Mm00515324_m1). mRNA expression levels are given as ratios to the glyceraldehyde-3-phosphate dehydrogenase (GAPDH).

***Statistical analysis***

All data were expressed as means±SD (n=6) for *in vivo* analysis in each group and compared using of one-way analysis of variance (ANOVA); values of *P*<0.05 were regarded as significant. For *in vitro *analysis, the Student test was done, values of *P*<0.05 were considered significant. SPSS software (version 28.0, Chicago, IL, USA) was used in all statistical analyses. 

## Results

***Renal function markers***

From the results represented in Table 1, CsA administration to rats for 14 days induced significant changes in kidney function evident by a significant (*P*<0.01) increase in serum creatinine and BUN compared to the control group. Treatment of animals exposed to toxic dose of CsA with EALD extract significantly (*P*<0.01) prevented the decrease of serum creatinine level by 77.14% and BUN level by 83.17% as well compared to Vit.E group. Moreover, The Vit.E+CsA group showed a significant protective effect by decreasing the creatinine level by 65.71 % and BUN level by 72.12% compared to the CsA group.

***Renal oxidative stress markers***

Administration of CSA to normal rats resulted in a significant (*P*<0.01) depletion of cortex renal antioxidant (SOD, GST, and GPX) activities and GSH level, accompanied by a significant (*P*<0.01) elevation in MDA level as compared with the control group (Table 2). In both (EALD+CsA) and (Vit.E+CsA) groups, there was a significant (*P*<0.01) reserve in SOD Activities (71.58%, 62.49%; *P*<0.01), in GST Activities (69.75%, 64.41%; *P*<0.01), in GPX Activities (85.64%, 77.60%; *P*<0.01), respectively in GSH level (78.57%, 69.20%; *P*<0.01), respectively and a significant (*P*<0.01) reserve of MDA content (87.80%, 73.71% ; *P*<0.01), respectively (Table 3).

***Kidney histology***

**Figure 1:**
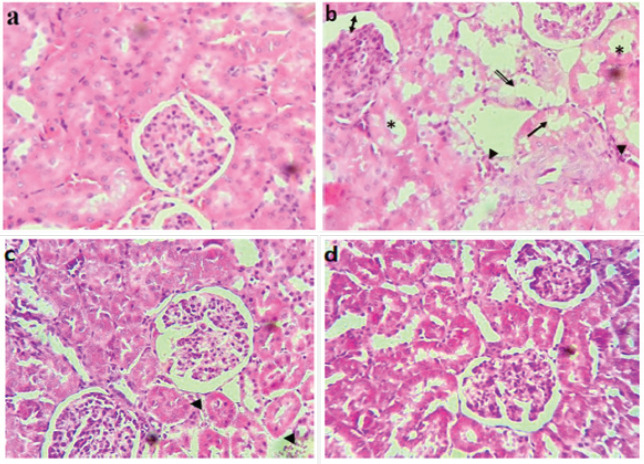
Photomicrograph of rat kidney sections (Hematoxylin-eosin, Magnification, x200)

The biochemical results were well supported by the results of the histopathological analysis, in this regard; Microscopic examination of renal tissue sections revealed no signs of tubular or glomerular damage in the control group (Figure 1A), while examination revealed severe changes in the histoarchitecture in kidneys of CsA-only-treated rat including tubular dilation and vacuolation, congestion of blood vessels, glomerular atrophy with thickening of the layers of Bowmen’s capsule (Figure 1B). On the other hand, EALD or Vit.E pretreatment of rats exposed to CsA administration showed a great improvement in the renal histological profile compared to the CsA group (Figure 1C, D). 

***Morphological changes of murine fibroblasts 3T3 cells after treatment with EALD extract (5 and 25*** ***μ*** ***g/ml) in combination with CsA 10*** ***μ*** ***g/ml***

**Figure 2:**
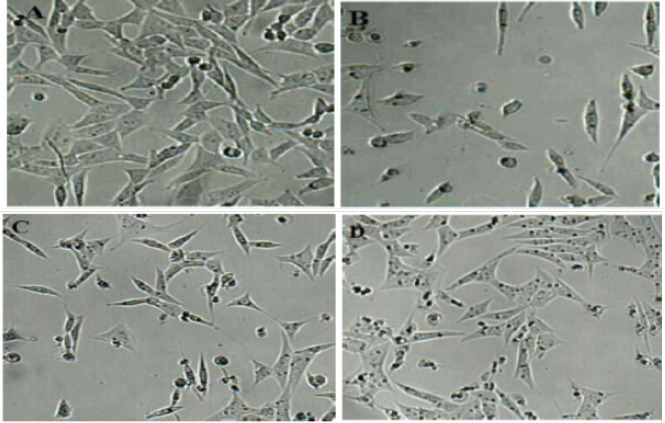
Microscopic images of morphological changes in murine fibroblasts 3T3 caused by CsA and effects of EALD extract 5 μg and 25 μg

As shown in Figure 2, large numbers of 3T3 cells cultured in CsA alone exhibited obvious changes in morphological appearance following disruption of the cytoskeletal structure, with cells becoming round or spindle-shaped with a marked decrease in the confluence (Figure 1B). these changes were clearly restored in a dose-dependent manner by EALD treatment (Figure 1C, D).

***ER Stress Markers***

*IRE1* *α* *expression by Immunohistochemistry*

**Figure 3:**
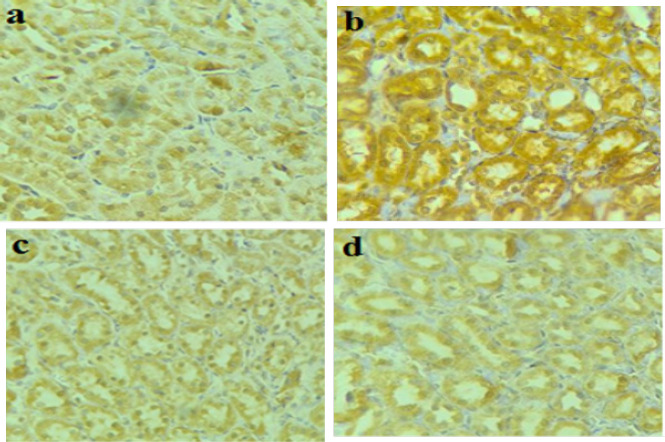
Representative immunohistochemistry of tubular IRE1α expression in rat kidney sections that were not subjected to treatment (control)

Tubular cells that expressed IRE1α positivity were characterized by a brownish-colored pigmentation in the cytoplasm around the nuclei (Figure 3). High staining was shown in the CsA group (b) that had positive expression of the IRE1α, compared to the other groups (Control, Vit.E+CsA, EALD+CsA)(a,c,d) which showed a low IRE1α expression.

***IRE1*** ***α*** ***expression by immunoblot***

**Figure 4:**
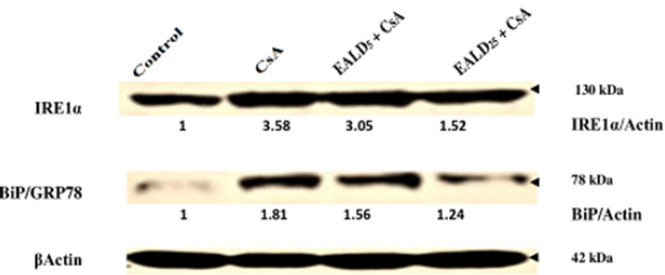
Representative immunoblot of ERS markers (IREα, BiP /GRP78 in murine fibroblasts 3T3 cells treated for 24 hr with 10 μg/ml CsA alone or with EALD extract (5 μg and 25 μg, respectively)

To explore ERS induced by CsA and the modulatory role of EALD in vitro; we detected the levels of ERS markers (IREα and BiP/GRP78) using murine fibroblasts 3T3 Cell lines. As shown in Figure 4, high levels of IRE1 and BiP proteins were observed in cells treated with CsA alone (IRE1/CsA Ratio=3.58, BiP/CsA Ratio=1.81). While cells treated with EALD extract (25 μg) showed a significant decrease in ER stress tested markers close to control cells (IRE1/Control Ratio=1.52, BiP /Control Ratio=1.24). These results support the potential abilities of the extract to regulate ERS during CsA cytotoxicity (Figure 4).

***Effect of L. duriusculum extract(EALD) on ER Stress gene expression (MCP-1, ATF4) in CsA cell treatments***

**Figure 5:**
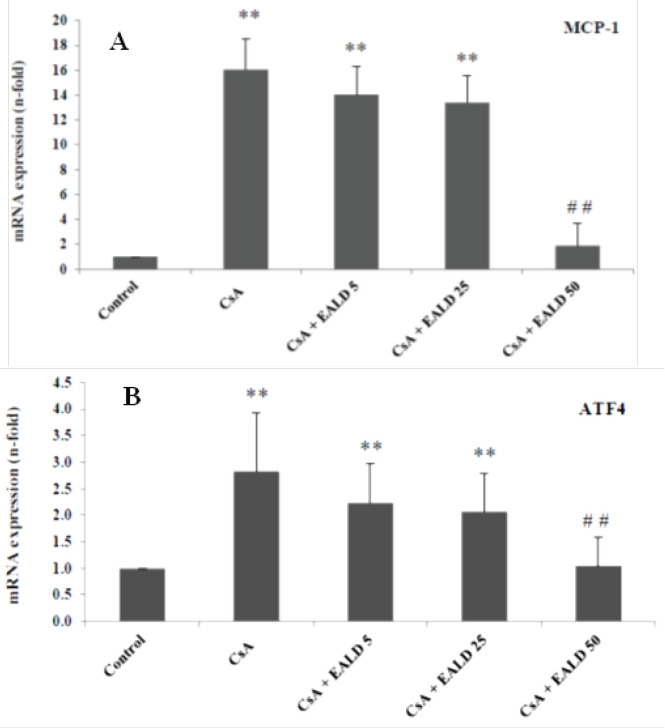
mRNA expression of MCP-1 (A) and UPR (PERK branch) member ATF4 (B) gene were evaluated by q-RT-PCR in MCT cells from control and cells incubated with CsA alone (10 μg/ml) or pretreated cells with various concentrations of EALD extract (5, 25, 50 μg/ml, respectively) for 6 hr

To evaluate whether EALD can decrease the transcription level of of ERS mediated by MCP-1 and ATF4,, MCT cells were used as in vitro model, qRT-PCR results were illustrated in Figure 5, MCP-1 mRNA expression was significantly (*P<*0.01) high in CsA-treated cells (800% of the control)(Figure 5A); suggesting elevated synthesis of proinflammatory cytokines in MCT cells, whereas pretreatment with a dose of 50 μg/ml EALD extract prevented CsA-induced inflammatory response by inhibiting MCP-mRNA transcription. 1 at this dose.

Figure 5B illustrates that CsA-induced ATF4 gene expression in MCT cells was significantly (*P<*0.01) attenuated by EALD by 50 μg/ml EALD treatment close to the gene expression of control cells; indicating inhibition of the unfolding protein response (UPR) contributing to CsA-induced ERS.

**Table 1:** Effect of *Limonium duriusculum* extracts (EALD)(200 mg/Kg) on renal function marker levels (Creatinine, BUN) in CsA (25 mg/kg) treated rats

Groups	Creatinin (mg/dl)	BUN (mg/dl)
Control	0.5±0.06	31.3±2.4
EALD	0.51±0.03	32.1±1.8
CsA	0.85±0.05*¥¥	84.8±5.75**¥¥
Vit.E+CsA	0.62±0.03£ (65.71 %)	45.8±3.9**¥¥££§§(72.12 %)
EALD+CsA	0.58±0.04£ (77.14 %)	40.3±3,5**¥¥££ (83.17 %)

Results are represented as means±SD, (n=6). Values in parentheses indicate the percentage of protection. The percent of protection is calculated as 100x (values of CsA)-values of samples/ (values of CsA-values of control)

*: Control vs all groups (*P<0.05, **P<0.01), ¥: EALD vs all groups except control (¥*P<*0.05, ¥¥*P<*0.01), £: CsA vs (Vit.E+CsA) and (EALD+CsA) (£*P<*0.05, ££*P<*0.01), §: (Vit.E+CsA) vs (EALD+CsA)(§*P<*0.05, §§*P<*0.01)

EALD: EtOAc fraction from L. duriusculum, CsA: Cyclosporine, Vit.E : Vitamine E

**Table 2:** IHC DAB concentration and the percentage of the stained area in the tubular cytoplasm (around the nuclei)

Groupes	MDA (μmol/mg tissue)	SOD (U/mg protein)	GSH (μmol/mg protein)	GST (U/mg protein)	GPx (U/mg protein)
Control	4.88±0.8	12.88±1.3	18.89±2.3	6.3±0.8	29.33±3.1
EALD	4.79±0.9	12.84±1.8	17.98±2.1	6.5±0.9	28.9±3.08
CsA	12.26±1.3**¥¥	7.32±0.9 **¥¥	9.18±1.1**¥¥	3.99±0.1**¥¥	11.78±1.2 **¥¥
Vit.E+CsA	6.82±0.9**¥¥££ (73.71%)	10.82±1.5**¥££ (62.49%)	15.95±2.01**¥££ (69.20%)	5.8±0.6££ (64.41%)	25.4±3.8**¥¥££ (77.6%)
EALD+CsA	5.78±0.2*¥££§ (87.80%)	11.3±1.3*¥££ (71.58%)	16.81±2.9*££ (78.57%)	5.95±0.3 ££ (69.75%)	26.81±4.1*¥ ££ (85.64%)

(0+) negative, (+) low positive, (++) positive, (+++) high positive

The brownish discoloration was increased in the CsA section compared to the control, indicating increased IRE1α expression in kidney tubular cells, while in both EALD+CsA and Vit.E+CsA groups the brownish discoloration appeared less brownish indicating the great inhibition of IRE1α expression. (IRE1α expression was quantified on brown-stained tissue sections with Image J 1.46 software (Wayne Rasband, National Institutes of Health, USA)

EALD: EtOAc fraction from L. duriusculum, CsA: Cyclosporine, Vit.E : Vitamine E, IHC : Immunohistochemistry, IRE1α: Inositol-requiring enzyme 1α

**Table 3:** Effect of *Limonium duriusculum* extract (EALD)(200 mg/Kg) on renal oxidative stress markers in CsA (25 mg/kg) treated rats

Image	Control	CsA	EALD+CsA	VE+CsA
IHC concentration	+	+++	++	++
Area %	9.52%	49.15%	13.82%	16.50%

Results are represented as means±SD, (n=6), Values in parentheses indicate the percentage of protection. The % of protection is calculated as 100 x (values of CsA)-values of samples/(values of CsA values of control). *: Control vs all groups (*P<0.05, **P<0.01), ¥: EALD vs all groups except control (¥*P<*0.05, ¥¥*P<*0.01), £: CsA vs (Vit.E+CsA) and (EALD+CsA)(£*P<*0.05, ££*P<*0.01), § : (Vit.E+CsA) vs (EALD+CsA)(§*P<*0.05, §§*P<*0.01)

MDA : Malondialdehyde; SOD : Superoxide dismutase; GSH : Reduced Glutathione; GST: Glutathione S-Transferase; GPx: Glutathion peroxydase; EALD: EtOAc fraction from L. duriusculum; CsA: Cyclosporine; Vit.E : Vitamine E

Kidney of control rats (a) showed a normal appearance of histological structures with regular morphology of glomerulus and tubules, whereas kidney sections from rats treated with CsA alone (b) revealed in various degrees in a glomerular and tubular blood vessels congestion and degenerative changes (double arrow), Tubular dilatation (Asterisk) and vacuolization (arrow). Glomerular atrophy and thickening of Bowmen’s capsule layers (double sided arrow) some hemorrhagic foci and inflammatory cells infiltration is also observed in the interstitium (arrowhead). The histopathological abnormalities seen in kidneys of rats treated with CsA alone are slightly present in both (Vit.E+CsA)(c), and (EALD+CsA) (d) Groups marked by hemorrhage (arrow head) and moderate diffuse vacuolations in renal tubules

EALD: EtOAc fraction from *L. duriusculum*, CsA: Cyclosporine, Vit.E : Vitamine E

3T3 cells were plated and grown as described in the materials and methods section. After 24 hr, we used phase-contrast microscopy at x16 to examine cells, then photographs were taken. A: Control, untreated cells, no changes were seen in the morphology of the cells. B: In cultures treated with CsA (10 μg/ml) alone, the majority of cells have lost their elongated appearances, and have become round in shape, others were long and fusiform. Moreover, the confluence of cells is lower than the control culture. C: Cells treated with EALD extract 5 μg in combination with CsA, revealed moderate changes in morphology and confluence. D: EALD extract 25 μg prevented clearly the morphological changes of 3T3 cells (Magnification, x400)

EALD: EtOAc fraction from *L. duriusculum*, CsA: Cyclosporine

(a), treated with CsA alone (b), treated with CsA and Vit. E (c), or treated with CsA and EALD (d). Images show a brownish-colored pigmentation in the tubular cytoplasm around the nuclei as evidence of CsA-induced IRE1α activation that was otherwise inhibited by Vit. E and EALD extract (c, d). (Magnification, ×200)

EALD: EtOAc fraction from *L. duriusculum*, CsA: Cyclosporine, Vit.E : Vitamine E, IRE1α: Inositol-requiring enzyme 1α

β-Actin was used as a loading control. Cell lines showing high levels of both IRE1 and BiP proteins in CsA alone treated cells. While EALD (25 μg) extract-treated cells showed a significant decrease in ER Stress tested markers close to the control cells. (IRE1/Control Ratio=1.52, BiP /Control Ratio=1.24)

EALD: EtOAc fraction from *L. duriusculum*, CsA: Cyclosporine, Vit.E : Vitamine E,

IRE1α: Inositol-requiring enzyme 1α, BiP : Binding immunoglobulin protein, GRP78: Glucose-regulated protein 78, ER : Endoplasmic reticulum

MCP 1: Inflammatory gene, (Monocyte chemotactic protein-1) Proinflammatory cytokine. ATF4: (Activating transcription factor 4), UPR (PERK branch) member. CsA induced ATF 4 gene expression in MCT cells, while the expression of this gene was significantly attenuated by EALD 50 μg/ml treatment close to the gene expression of control cells.

The expression of MCP-1 mRNA was significantly elevated in CsA-treated cells (800% of the control). Pretreatment with a dose of 50 μg/ml of EALD extract prevents the inflammatory response induced by 10 μg/ml CsA in MCT cells treated for 6 hr by inhibiting MCP-1 mRNA transcription at this dose

Bar graphs represent the mean±SD of three independent experiments. *P<0.05, **P<0.01 versus control; #*P<*0.05, ##*P<*0.01 versus CsA

CsA: Cyclosporine, EALD: EtOAc fraction from *L. duriusculum*, MCP1: Monocyte chemoattractant protein-1, UPR: unfolded protein response, ATF4: Activating transcription factor 4

## Discussion

Several studies attempted to express the molecular mechanism of CsA-induced renal toxicity. Oxidative/endoplasmic reticulum stress and inflammation in mesangial, endothelial, and tubular cells during renal injury have been suggested to play an important role (31) in which oxygen radicals can disrupt the structural integrity of cell and organelle membranes and their functions and metabolic processes (32). Therefore, the present study focused on maintaining renal redox homeostasis through the modulatory effect of EALD on ROS/ERS in rat kidneys.

The present study investigated the potential renal protective effects of EALD on rat kidneys exposed to CsA. The results indicated that the group of animals treated with CsA for fourteen days exhibited significant renal impairment, as evidenced by elevated levels of urea and serum creatinine. This impairment is likely attributed to a decrease in glomerular filtration rates and renal blood flow, resulting from the vasoconstrictor effect of CsA (33). Histopathological testing supported these findings, revealing extensive renal tubular injury. The findings were consistent with prior research demonstrating substantial renal impairment in patients and experimental animals following CsA treatment (33, 12). On the other hand, when rats were dosed with *L. duriusculum *extract (200 mg/kg/day) or vitamin E (100 mg/kg) for fourteen days before CsA administration, their vulnerability to kidney impairment was reduced. While various studies have attempted to elucidate the molecular mechanisms of CsA-induced renal toxicity, a complete understanding of this mechanism remains elusive. Nevertheless, oxidative stress and inflammation have been proposed to play a crucial role (34, 11, 12).

In fact, the elevated level of TBARS detected in the renal tissues of rats exposed to CsA (25 mg/kg) in the current investigation indicated a significant degree of lipid peroxidation and oxidative stress. Moreover, lipid peroxidation in endothelial and mesangial cells was found as a significant consequence of the alteration of oxidative status during CSA cytotoxicity, manifested by an increase in the production of aldehydes, in particular, the essential product, malondialdehyde (34) wherein oxygen radicals can disrupt the structural integrity of cell and organelle membranes, affecting their functions and metabolic processes (35). 

Our investigation also noted occurrences related to MDA production, including a decline in the activities of the renal cortex SOD, GPx, and GST, along with a depletion of GSH. SOD, an essential antioxidative enzyme, plays a pivotal role in catalyzing the dismutation of superoxide anion into hydrogen peroxide and molecular oxygen (36). The decrease in SOD activity observed in CsA-treated groups may be due to the scavenging of several ROS generated during CsA metabolism. However, the reduction of GPx and GST activities suggests a decrease in their substrate and GSH levels coupled with an elevation in peroxide levels. A clear correlation indeed was observed between GSH depletion and CsA-induced ROS generation. 

It is worth highlighting that CsA-induced ROS generation may also lead to increased leakage of Ca^2+^ from the ER lumen. Elevated cytosolic Ca^2+^ levels can significantly trigger mitochondrial ROS production, subsequently inducing pore opening allowing GSH to leak out of the matrix (37). Notably, GSH, a major non-protein thiol found in all cell types, is primarily synthesized in the liver and serves as a regulator of intracellular redox homeostasis (38). The activity of GPx is controlled by intracellular hydrogen peroxide (H_2_O_2_) levels, which in turn regulate lipid peroxidation and contribute to maintaining the balance of ROS (39).

In contrast, GST forms conjugates with glutathione, neutralizing toxic electrophilic metabolites generated in the cell through the catalytic actions of cytochromes P-450 phase 1 (40). The cytoprotective effect of EALD and Vitamin E was associated with the preservation of GSH, along with increased levels of GPx and SOD.

Our findings align with other studies that have reported an inhibitory effect of the *Limonium* genus on lipid peroxidation (41) and its ability to preserve Ca^2+^ homeostasis (42). EALD treatment significantly decreased lipid peroxidation and structural kidney damage in CsA-treated groups, likely due to the antioxidant properties of *Limonium* as highlighted in our prior study (17). This effect is reminiscent of vitamin E, known for acting as a chain-breaking antioxidant by inhibiting excessive lipid peroxidation (43). The antioxidant potency and renoprotective properties of EALD may be attributed to its high content of phenolic and flavonoid compounds, in particular apigenin, or most likely to the synergistic interactions among these compounds referenced in our previous phytochemical study (17). These findings are consistent with recent reports showing the effectiveness of phenolic compounds of the genus *Limonium, *such as apigenin, quercetin, kaempferol, and myricetin, against oxidative stress and inflammatory responses in various cell types (44, 21).

There is a clear connection between oxidative stress and ERS, establishing a cause-and-effect relationship with each other. It has been reported that oxidative stress can trigger and contribute to ERS (45). Whereas ER provides an oxidizing environment that promotes the formation of disulfide bonds formation, this process likely accounts for up to 25% of total ROS production (46, 37). Chronic activation of ERS and UPR pathways in endothelial cells leads to increased oxidative stress and inflammation and often results in cell death (47) which contributes to the pathogenesis of many diseases including the development and progression of kidney disease (48). In this context, ERS can induce severe cellular damage and lead to renal fibrosis and apoptosis (49). These cellular abnormalities due to the increased ERS may often be associated with kidney damage with a significant increase in the proteinuria level, which in turn leads to glomerular filtration barrier defects; resulting in an increased glomerular permeability and various renal disorders (50). Indeed, studies on CsA revealed a close relationship between ERS and UPR in renal tubular cells as part of the general inflammatory response (51).

UPR signaling cascades are initiated via three ER membrane proteins: IRE1α, pancreatic ER kinase (PERK), and activating transcription factor 6(ATF6), and are tightly interrelated with inflammatory pathways (52).

To explore whether EALD targeting influences CsA-induced renal UPR activation. Our current study assessed ERS levels in CsA- renal tissues and cells by assessing the expression of three UPR molecules: IRE1α, activating transcription factor 4 (ATF4)(a downstream factor of PERK), and glucose-regulated protein 78 (GRP78) also referred to as BiP (a central regulator of ER function). Kidney immunohistochemistry analysis showed that CsA induced the redistribution of IRE1α in the perinuclear cytoplasm of tubular cells, indicating IRE1α activation. IRE1α signaling is an adaptive cellular response, but under high levels of ER stress, it has been shown to become cytotoxic and contribute to the activation or up-regulation of various pro-inflammatory proteins and pro-apoptotic agents (53). Immunohisto-analysis results also revealed that co-treatment with EALD down-regulated the IRE1α expression in tubular rats’ kidney cells. Furthermore, *in vitro* experiments revealed that EALD pretreatment significantly reduced the IRE1α response in murine fibroblasts 3T3 cells, confirmed by immunoblot analysis. This reduction was followed by less expression of BiP/GRP78 indicating the possible ability of EALD to resist CsA-induced ER stress. Regarding PERK, one of the stress kinases, when activated in response to a wide variety of stressors, it phosphorylates eukaryotic initiation factor 2 protein (eIF2) and consequently inhibits protein synthesis, preventing further increases in protein-folding demand in the ER (54). Phosphorylated eIF2α can also increase the expression of ATF4, which may also enhance the expression of UPR target genes associated with oxidative stress, inflammation, and apoptosis under chronic ER stress (54, 53 ). ATF4 promotes the expression of genes related to amino acid transport and glutathione biosynthesis (55) and has been involved in regulating redox balance, playing a part in protecting fibroblasts, hepatocytes, and tubular cells from oxidative stress and toxicity (55-57). In addition, ATF4 is crucial in controlling the regulation of pro-apoptotic transcription factor CHOP and GRP78 expression (57). Whereas the induction of CHOP could be a negative side effect of any stress stimuli that increases ATF4 levels (58).

Our results demonstrated that a mechanism underlies the inhibitory effect of EALD (50 μg/ml) on ER stress mediated by elevated ATF4 expression in murine proximal tubular epithelial (MCT) cell line pretreated with CsA (10 μg/ml), this mechanism remains unclear. However, our findings support the possibility that EALD extract modulates the PERK-eIF2α-ATF4 pathway in CsA-activated UPR through its antioxidant properties. Antioxidants have already been shown to reduce ER stress (59). On the other hand, recent research reveals a cross-talk at multiple levels between ER stress, the UPR, and inflammation. Therefore, it focuses on determining the role of ER stress within certain cells and tissues involved in the inflammatory and immunological responses (60, 61). These complex interconnections vary depending on the metabolic status of cell type and specific condition, significantly increasing inflammatory mediators (61). IRE1α has been reported to be significantly involved in inflammation and immunity via their RNase and kinase domains (62), leading to the regulation of gene expression of proinflammatory cytokines such as IL-6, IL-8, TNF-α, and MCP1 (chemoattractant protein 1), associated with the activation of the NF-κB (nuclear factor-kappa B) pathway in different inflammatory disorders, for example in skin inflammation (63), during viral myocarditis (64), and in renal tubule cells under metabolic stress (65). Moreover, both spliced XBP1(X-box binding protein 1) as adown stream target of IRE1 and ATF4 also as transcription factors of UPR may be involved in regulating the production of several inflammatory cytokines/chemokines including MCP1 in endothelial cells; such as aortic cells (66) microvascular endothelial cells of the brain and retina (67). Whereas ATF4 inhibition had a similar inhibitory effect on MCP1 expression (66). MCP1/CCL2 is an essential member of the CC chemokine family that controls the migration and infiltration of monocytes and macrophages to inflamed tissue sites (68) as well as regulating gene transcription implicated in cell death or differentiation (52, 56). Additionally, MCP-1 is one of the signaling mechanisms linking ER stress and inflammation (52). According to various studies, elevated levels of MCP-1 have been observed in chronic inflammation associated with oxidative stress in various diseases and disorders such as liver injury, kidney during ischemia/reperfusion injury, and diabetic nephropathy in both Type 1 and Type 2 (68). In this context and given the above-mentioned promising results of EALD extract in modulating CsA-induced ERS, we are encouraged to verify the anti-inflammatory effect of EALD on the MCP-1 marker in tubular cells. qRT-PCR analysis demonstrated that MCP-1 expression was markedly increased in MCT cells treated with CsA alone, while a dose of 50 µg/L of EALD extract significantly decreased the expression level of MCP-1 close to control cells; thus, these data improve the ability of EALD extract to reduce an important pro-inflammatory promoter such as MCP-1 in cultured renal tubular cells treated with CsA. Our study demonstrated that EALD extract could improve renal damage inhibition by reducing both ERS and inflammation in rat kidney and cell culture models.

 In this context, pharmacological and natural inhibitors and inducers of ERS were tested to better understand the implication and importance of UPR in various experimental disease models (59). Several findings suggested that modulating ERS or inhibiting molecular chaperones during UPR may provide new therapeutic approaches for kidney injury (69). In addition to the chemical products, natural compounds such as certain polyphenols have been to attenuate the protein expression involved in UPR signaling, such as PERK-ATF4-CHOP, BiP/GRP78, and IRE1-XBP1, and consequently ER stress-related apoptosis in various renal disorders (6). Over recent years an increasing number of plant metabolites have been investigated such as resveratrol (10), quercetin (70), and ginsenoside-Rg1 (71). In this regard, numerous studies have indicated the effective and beneficial role of apigenin in modulating renal injury accompanied by ERS (72, 73). Our previous phytochemical study revealed the presence of numerous bioactives including the major compounds apigenin and apigenin 7-O-b-D-(6’’-methylglucuronide) (17). Our results support the possibility that EALD extract modulates the PERK-eIF2α-ATF4 pathway in the CsA-activated UPR through its antioxidant properties. These data improve the ability of EALD extract to reduce an important pro-inflammatory promoter such as MCP-1 in cultured renal tubular cells treated by CsA. Our study demonstrated that EALD extract could improve renal damage inhibition by reducing both ERS and inflammation in rat kidney and cell culture models; natural compounds such as certain polyphenols have been able to attenuate the protein expression involved in UPR signaling, such as PERK-ATF4-CHOP, BiP/GRP78, and IRE1-XBP1, and consequently ER stress-related apoptosis in various renal disorders, numerous studies have indicated the effective and beneficial role of apigenin in modulating renal injury accompanied by ERS (72, 73). Our previous phytochemical study revealed the presence of numerous bioactives including the major compounds apigenin and apigenin 7-O-b-D-(6’’-methylglucuronide)(17).

Given the promising results mentioned above on the renoprotective effect of EALD extract which was exerted for the first time by various modulatory mechanisms, suppression of ROS generation in the CsA rat model, modulation of ERS (ATF4, IRE1α, BiP), and restriction of inflammation (MCP1) on 3T3 cells and MCT cell culture, we hypothesized that EALD extract successfully maintained the renal redox homeostasis, which could be due to synergistic interactions of apigenin with other EALD compounds.

## Conclusion

Collectively, these findings suggest that EALD could potentially protect the kidneys against CSA toxicity. In this context multiple mechanisms are proposed to contribute to the understanding of the beneficial effects of ethyl acetate extract of *L. duriusculum *such as alleviation of oxidative stress by decreasing MDA and enhancing GSH level, GST, GPX, and SOD activities, modulation of ERS via down-regulation of IRE1α, BiP, and UPR member ATF4 (PERK branch), and preventing the inflammatory response induced by CSA (MCP-1 down-regulation).

These results were proven by immunohistochemical staining, immunoblot analysis, and qRT-PCR analysis. Further research is necessary to investigate the effect of *L. duriusculum *and its compounds on more UPR pathways.

## Authors’ Contributions

RS A, S A, A O, and B K designed the experiments; RS A, M K, K W, and AM R performed experiments and collected data; RS A, S A, and A M discussed the results and strategy; S A supervised, directed, and managed the study; S A and A M approved the final version to be published.

## Conflicts of Interest

The authors report no potential conflicts of interest.
